# A Systematic Review on Comparative Analyses between Ureteroscopic Lithotripsy and Shock-Wave Lithotripsy for Ureter Stone According to Stone Size

**DOI:** 10.3390/medicina57121369

**Published:** 2021-12-16

**Authors:** Hae Do Jung, Youna Hong, Joo Yong Lee, Seon Heui Lee

**Affiliations:** 1 Department of Urology, Wonkwang University Sanbon Hospital, Wonkwang University College of Medicine, Gunpo 15865, Korea; haedojung79@wku.ac.kr; 2 Division of New Health Technology Assessment, National Evidence-Based Healthcare Collaborating Agency, Seoul 04554, Korea; yunahong@neca.re.kr; 3 Department of Medical Device Management and Research, Yonsei University College of Medicine, Seoul 03722, Korea; 4 Department of Urology, Severance Hospital, Urological Science Institute, Yonsei University College of Medicine, Seoul 03722, Korea; 5 Center of Evidence Based Medicine, Institute of Convergence Science Yonsei University, Seoul 03722, Korea; 6 Department of Nursing Science, College of Nursing, Gachon University, Incheon 22212, Korea

**Keywords:** ureteral calculi, lithotripsy, ureteroscopy, systematic review, meta-analysis

## Abstract

*Background and Objectives:* This systematic review and meta-analysis was conducted to analyze the treatment outcomes of shock wave lithotripsy (SWL) and ureteroscopic lithotripsy (URSL) according to the ureteral stone size. *Materials and Methods:* In this systematic review, relevant articles that compared SWL and URSL for treatment of ureteral stones were identified. Articles were selected from four English databases including Ovid-Medline, Ovid-EMBASE, the Cochrane Central Register of controlled Trials (Central), and Google Scholar. A quality assessment was carried out by our researchers independently using the Scottish Intercollegiate Guidelines Network (SIGN). A total of 1325 studies were identified, but after removing duplicates, there remained 733 studies. Of these studies, 439 were excluded, 294 were screened, and 18 met the study eligibility criteria. *Results*: In randomized control trial (RCT) studies, URSL showed significantly higher SFR than SWL (*p* < 0.01, OR= 0.40, 95% CI 0.30–0.55, I² = 29%). The same results were shown in sub-group analysis according to the size of the stone (<1 cm: *p* < 0.01, OR = 0.40, 95% CI 0.25–0.63; >1 cm: *p* < 0.01, OR = 0.38, 95% CI 0.19–0.74, I² = 55%; not specified: *p* < 0.01, OR = 0.43, 95% CI 0.25–0.72, I² = 70%). In the non-RCT studies, the effectiveness of the URSL was significantly superior to that of SWL (*p* < 0.01, OR = 0.33, 95% CI 0.21–0.52, I² = 83%). Retreatment rate was significantly lower in URSL than in SWL regardless of stone size (*p* < 0.01, OR = 10.22, 95% CI 6.76–15.43, I² = 54%). *Conclusions:* Meta-analysis results show that SFR was higher than SWL in URSL and that URSL was superior to SWL in retreatment rate. However, more randomized trials are required to identify definitive conclusions.

## 1. Introduction

Urolithiasis is a challenging worldwide disease with high reported prevalence rates of 7–13% in the United States and Canada and 5–9% in European countries, but only 1–5% in Asian population [1]. Important elements that may influence the formation of ureter and renal stones include increases in diagnosis of co-morbidities such as metabolic syndrome, changes of life cycle, and dehydration status, including water intake and urine volume [2]. Recently, several studies have indicated that seasonal changes, particularly during the hot season, and global warming also have impacts on the increase in renal stones on a global scale [3]. The predicted increment in urolithiasis is by 1.6–2.2 million lives by the year 2050, which will increase health care expenditures by 25% [1].

Shock wave lithotripsy (SWL) is a minimally invasive treatment modality in urinary tract stone disease, but when the size of the stone is large, the success rate of SWL is low and multiple treatments may be required. Ureteroscopic lithotripsy (URSL) was used in its early stages of development as a treatment for lower ureter calculus, but now it also has a high success rate in treating upper ureter stones due to the improved technology of endoscopes, which include videoscopes and disposable flexible ureteroscopes [4].

The European Association of Urology guidelines consider ureteral stone location and size as factors in guiding the modality of treatment. Guidelines recommend SWL for proximal ureteric stones measuring < 10 mm and either SWL or URSL as valid options for stones that are > 10 mm. In cases of distal ureteric stones, the guidelines favor URSL for stones > 10 mm, and either URSL or SWL for stones < 10 mm [5]. However, in real clinical settings, urologists can decide between the URSL and SWL, considering their available armamentarium and area of expertise in each case regardless of the recommendations of several guidelines [6]. Thus, the purpose of the study was to analyze the treatment outcomes of SWL and URSL according to the size of the stone. Our hypothesis was that URSL would be superior to SWL.

## 2. Materials and Methods

### 2.1. Inclusion Criteria

The inclusion criteria of this study were as follows: (a) ureteral stone patients, (b) comparison intervention between SWL and URSL, and (c) outcome measures including stone-free rate (SFR). A published study was excluded if it was not a full-text or only an abstract. This systematic review and meta-analysis were performed according to the standard PRISMA (Preferred Reporting Items for Systematic Reviews and Meta-Analyses) statement (Supplement Table S1) [7]. This systematic review was exempt from review by the ethics committee or institutional review board because systematic reviews and meta-analyses do not require ethical approval.

### 2.2. Search Strategy

A systematic review was carried out to identify relevant comparative articles between SWL and URSL for ureteral stone using Ovid-Medline (1946–October 2021), Ovid-EMBASE (1974–October 2021), the Cochrane Central Register of controlled Trials (Central), and Google Scholar.

A search strategies were established to include Medical Subject Headings (MeSH), keywords and search terms including as “ureterolithiasis”, “urolithiasis”, “ureter stone”, “shock wave lithotripsy”, “SWL”, “extracorporeal shock wave lithotripsy”, “ESWL”, “lithotripsy”, “ureteroscopic lithotripsy”, “flexible ureteroscope”, “URSL”, and combinations of search terms.

### 2.3. Study Selection and Extraction

Our researchers screened titles and abstracts independently identified by the search strategy to exclude irrelevant studies. They also assessed the full-text of the articles to conduct a search for potentially relevant articles. The most relevant articles were extracted from each study, and the author, year of publication, country, study design, patient characteristic participants (e.g., age, stone size), treatments, etc., were recorded.

Our researchers also extracted outcome variables such as “stone free rate” and “retreatment procedure”, wrote them in a data extraction file, and double-checked those variables.

### 2.4. Quality Assessment

We used the Cochrane Risk of Bias (ROB) tool for randomized control trials (RCTs) and the methodological index for nonrandomized studies (MINORS) for nonrandomized studies. We graded the quality of evidence using the Scottish Intercollegiate Guidelines Network (SIGN), composed of various types of research, including systematic reviews and meta-analyses, randomized controlled trials, cohort studies, case–control studies, diagnostic studies, and economic studies. A quality assessment was carried out by our researchers independently (Y.H. and H.D.J.). All disagreements of the quality assessment results were cleared up after discussion with a third reviewer (J.Y.L.).

### 2.5. Statistical Analysis

The odds ratios (ORs) and 95% confidence intervals (Cis) were calculated and reported. The chi square test with *p* values less than 0.05 was used to evaluate statistical heterogeneity, and *I*^2^ was used to quantify heterogeneity [8]. If reported *I*^2^ was less than 50%, we applied the fixed effect model; otherwise, the random effect model was applied. Higgins *I*^2^ was calculated as below:

$$I^{2}=\frac{Q-df}{Q}\times 100\%$$

where *Q* is the Cochrane heterogeneity statistic and *df* is degrees of freedom. All meta-analyses was performed using Review Manager, Version 5.3 (RevMan, Copenhagen: The Nordic Cochrane Center, The Cochrane Collaboration, 2013).

Sub-group analysis was also performed in three groups according to the size of the stone. The sizes of stones were classified as ≤1 cm, ≥1 cm, or ‘not specified’ if the sizes of stones were not distinguished. This systematic review is registered in PROSPERO, CRD42021297311.

## 3. Results

### 3.1. Eligible Studies

**Figure 1:**
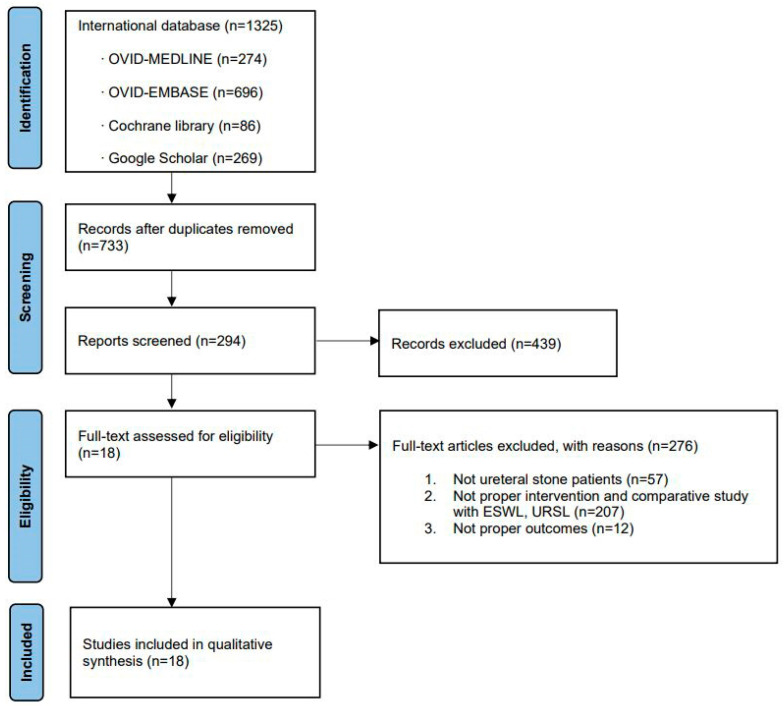
Study flow chart.

After reviewing all the original texts, 18 articles were identified as relevant for current meta-analysis (Figure 1) [9,10,11,12,13,14,15,16,17,18,19,20,21,22,23,24,25,26].

### 3.2. Characteristics of Included Studies with Quality Assessment and Publication Bias

**Figure 2:**
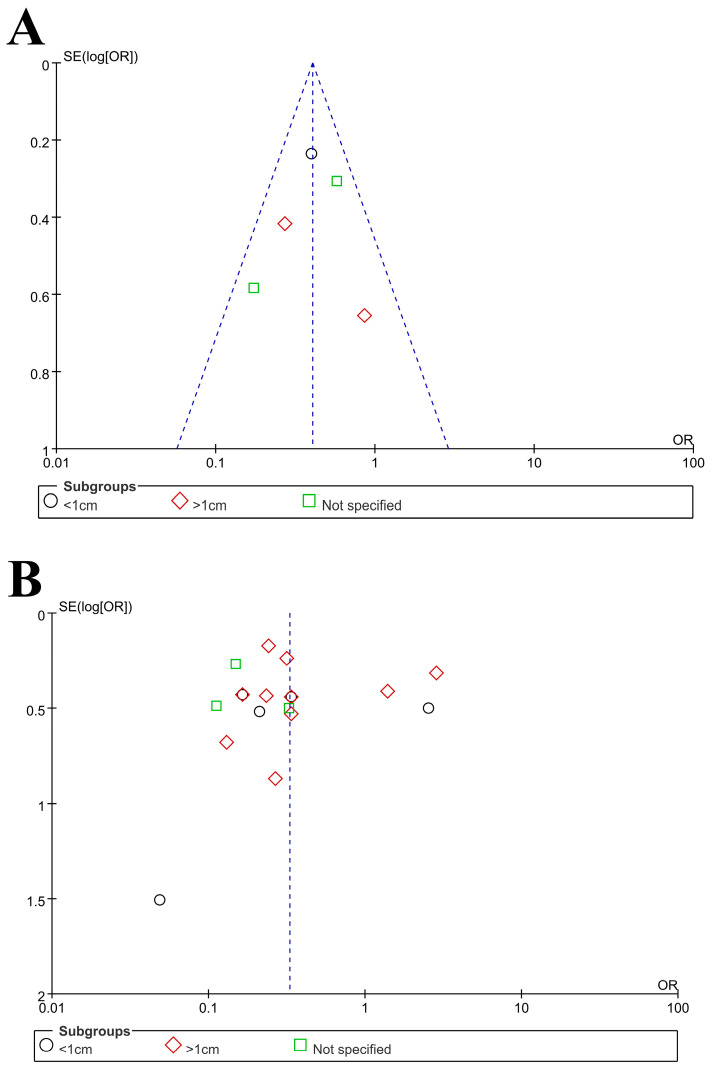

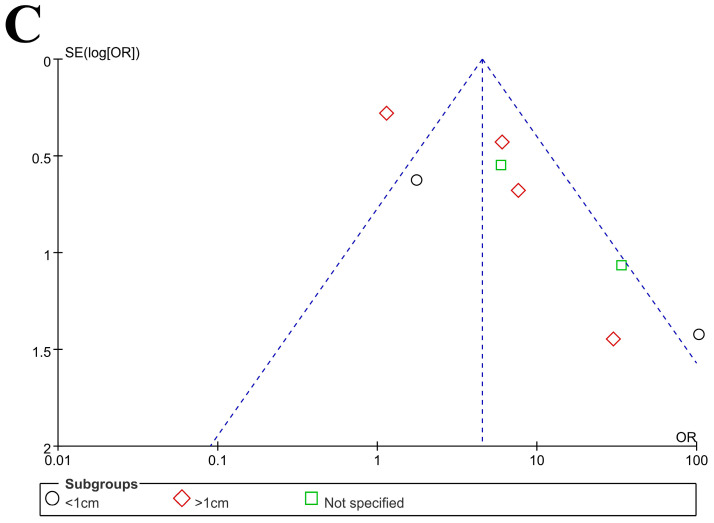
Funnel plot: (**A**) Stone-free rate (SFR) in randomized controlled trials (RCTs), (**B**) SFR in non-RCTs, (**C**) Retreatment.

**Figure 3:**
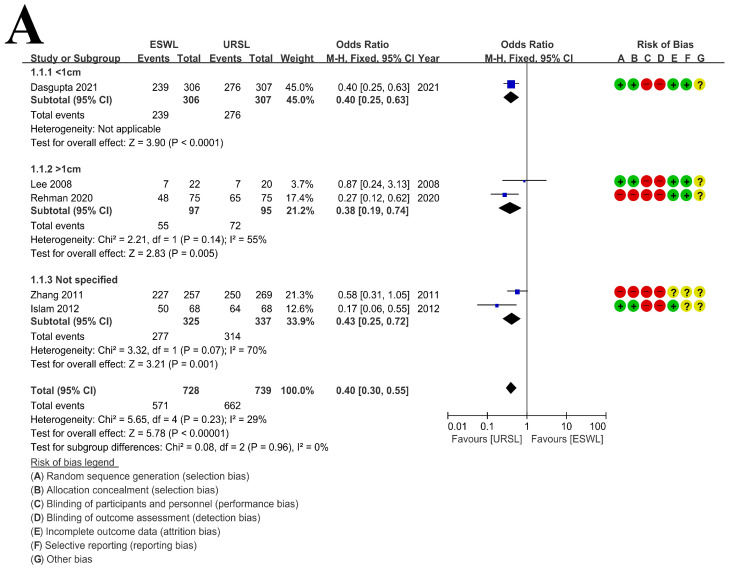

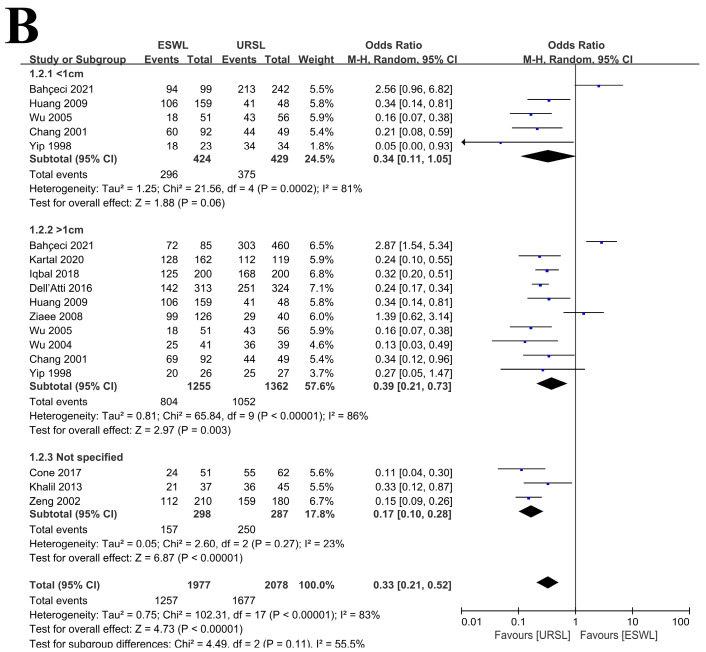
Meta-analysis: (**A**) SFR in RCTs and (**B**) SFR in non-RCTs.

The characteristics of the 18 included studies are shown in Table 1 [9,10,11,12,13,14,15,16,17,18,19,20,21,22,23,24,25,26]. All these comparative studies included patients who underwent URSL or SWL for ureteral stone treatment. The included studies were published from July 1998 to September 2021. Twelve of the eighteen studies were performed in Asia (Taiwan, China, Singapore, Kuwait, Iran, and Pakistan) [9,10,11,14,15,16,17,18,19,20,21,24], two in the USA [12,26], and two in Turkey [22,25], and one study was performed in Italy [13] and one the UK [23]. The included studies were divided on the basis of 1 cm stone size [11,13,15,16,17,18,20,21,22,23,24,25,26], and five studies failed to divide into 1 cm stone size [9,10,12,14,19]. The quality assessment results using SIGN of the included studies are shown in Table 1. Funnel plots of these meta-analyses are shown in Figure 2. Most of the included studies were located in the funnel. The ROB for five RCTs is displayed in Figure 3A. The MINORS scores for all the nonrandomized studies are displayed in Table 2. All studies were reasonable. 

### 3.3. Heterogeneity Assessment

**Figure 4:**
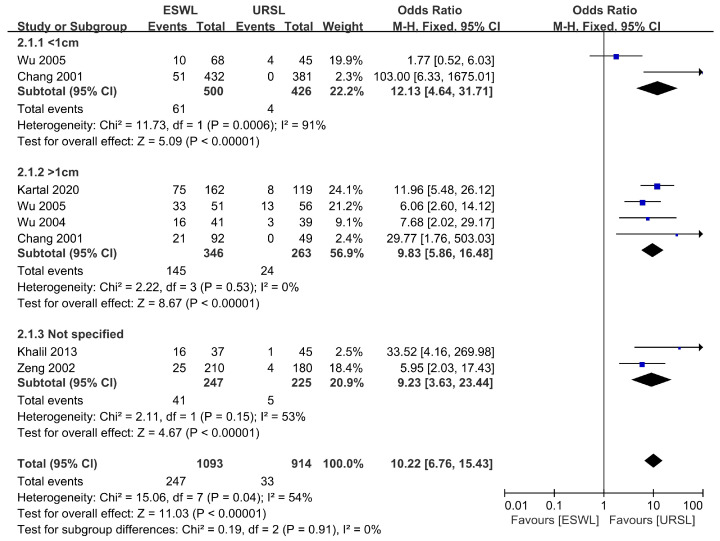
Meta-analysis: Retreatment procedure.

A heterogeneity test showed little heterogeneity in the analysis for SFR in RCT, so fixed effect models were fulfilled (Figure 3A Forest plot). However, conspicuous heterogeneities were discerned in the analysis for SFR in non-RCT (Figure 3B Forest plot), so random-effects models were used to compare SFRs between two treatments. There was little heterogeneity in the analysis of retreatment procedures, so fixed effect models were fulfilled (Figure 4 Forest plot).

### 3.4. Stone-Free Rate

Five studies were included for SFR in RCT [9,10,11,23,26]. Stone size in only one study was <1 cm [23], in two studies was > 1 cm [11,26], and was not specified others [14,19]. In RCT studies, URSL showed significantly higher SFR than SWL (*p* < 0.01, OR = 0.40, 95% CI 0.30–0.55, I² = 29%). The same results were shown in sub-group analysis according to the size of the stone (<1 cm: *p* < 0.01, OR = 0.40, 95% CI 0.25–0.63; >1 cm: *p* < 0.01, OR = 0.38, 95% CI 0.19–0.74, I² = 55%; not specified: *p* < 0.01, OR = 0.43, 95% CI 0.25–0.72, I² = 70%).

Thirteen studies were included for SFR in the non-RCT group [12,13,14,15,16,17,18,19,20,21,22,24,25]. The studies were divided according to stone size, namely <1 cm, ≥1 cm, and not specified. The effectiveness of URSL was superior to SWL (*p* < 0.01, OR = 0.33, 95% CI 0.21–0.52, I² = 83%). In sub-group analysis, URSL also showed significantly higher SFR than SWL in >1 cm, and not specified group (>1 cm: *p* < 0.01, OR = 0.39, 95% CI 0.21–0.73, I² = 86%; not specified: *p* < 0.01, OR = 0.17, 95% CI 0.10–0.28, I² = 23%). However, there was a tendency for URSL to have a better SFR than SWL in the <1 cm group, but it did not reach statistical significance (*p* = 0.06, OR = 0.34, 95% CI 0.11–1.05, I² = 81%).

The heterogeneity of studies in non-RCT was high. The reason was due to there being just one heterogeneous study [22]. In a meta-analysis of the non-RCT studies excluding the heterogeneous study, the heterogeneity was reduced to 50%. Additionally, in sub-group analysis excluding the heterogeneous study, the heterogeneity was reduced to 0% in the <1 cm group and 58% in the >1 cm group, respectively. However, we included the heterogeneous study because it was important due to there being few studies favoring SWL. 

### 3.5. Retreatment Procedure

Six comparative studies were included for the retreatment procedure, and sub-group analysis was conducted according to stone size. The three sub-groups were <1 cm, ≥1 cm, and not specified [14,17,18,19,20,25]. Retreatment rate was significantly lower in URSL than in SWL (*p* < 0.01, OR = 10.22, 95% CI 6.76–15.43, I² = 54%). In all sub-group analyses, URSL had fewer retreatment procedures than SWL (<1 cm: *p* < 0.01, OR = 12.13, 95% CI 4.64–31.71, I² = 91%; >1 cm: *p* < 0.01, OR = 9.83, 95% CI 5.86–16.48, I² = 0%; not specified: *p* < 0.01, OR = 9.23, 95% CI 3.63–23.44, I² = 53%, respectively).

A RCT study of retreatment procedures [9], which was not included for analysis, reported that 13 of 68 patients in the SWL group and 4 of 68 in the URSL group required retreatment.

## 4. Discussion

The principal treatments for ureteral stones are considered to be URSL and SWL. Chaussy performed SWL on patients for the first time in 1980 [27]. Subsequently, there has been further technique development of SWL and continuous modification of lithotripsy devices. However, the role and use of SWL has been declining due to the development of endourologic treatments, for example, URSL and percutaneous nephrolithotomy (PCNL) [28].

In 1912, Hugh Hampton Young performed ureteroscopy for the first time [29]. Although a rigid pediatric cystoscope, he identified a seriously hydroureter in a pediatric patient with posterior urethral valves. The first flexible ureteroscopy was performed in 1964 by Marshall who placed a 9 Fr flexible fiber-optic scope into a ureter to inspect stone during an open ureterotomy [30]. Recent advances in new digital flexible ureteroscopes, smaller nephroscopes, and holmium:yttrium–aluminum–garnet (Holmium: YAG) lasers have contributed to the surgical development of flexible URSL and PCNL [31]. However, SWL is still recommended by major guidelines as an essential treatment method for ureteral stones [5,32]. Furthermore, there is a controversy about URSL and SWL regarding the proper management of ureteral stones.

The advantage of SWL is that it shows fewer complications because it is non-invasive. However, in cases of severe obesity and mid or distal ureteral stones that require intervention, URSL is the first-line therapy [5,32]. The advantages of URSL are a greater stone-free rate in a single session and fewer retreatments. The disadvantages of URSL are associated with additional procedures, higher complication rates, and more extended hospital stays [5,32]. However, the advance of smaller caliber ureteroscopes and flexible ureteroscopes combined with Holmium:YAG laser has increased the stone-free rates and reduced the risk of complications [33].

Xu et al. studied a meta-analysis of the efficacy of URSL and SWL on ureteral calculi. They enrolled 13 papers and reported a SFR, repeat treatment rate, patient satisfaction, postoperative complications, operation time, and hospital stays [34]. They demonstrated that SWL showed a lower SFR (*p* < 0.001, RR 0.82, 95% CI 0.74–0.90), a higher repeat treatment rate (*p* = 0.004, RR 3.46, 95% CI 1.50 to 7.97), lower patient satisfaction (*p* = 0.02, RR 0.87, 95% CI 0.78–0.98), lower postoperative complications (*p* = 0.40, RR 0.63, 95% CI 0.48 to 0.83), and shorter operation times (*p* = 0.002, SMD −1.12, 95% CI −1.81–−0.43) and hospital stays (*p* = 0.004, SMD −1.71, 95% CI −2.88–−0.55) than URSL. However, the authors did not analyze by stone size [34].

Cui et al. reported a meta-analysis comparing SWL and URSL for treating large proximal ureteral stones [35]. They excluded studies in which the mean stone length was < 1 cm, and ultimately, 10 studies were analyzed for SFR, retreatment rate, operation time, auxiliary procedure rate, and complication rate between SWL and URSL. The results show that URSL was superior to SWL on SFR (*p* = 0.001, OR 0.349, 95% CI 0.183–0.666), and retreatment rate (*p* < 0.001, OR 7.192, 95% CI 4.934–10.482), whereas the operating time (*p* = 0.056, difference in mean of operation times = 10.35, 95% CI −0.29–20.99), complication rate (*p* = 0.598, OR 0.78, 95% CI 0.304–1.984), and the rates of auxiliary procedures (*p* = 0.929, OR 1.043, 95% CI 0.415–2.616) were not significantly different between SWL and URSL. These researchers concluded that URSL should be the treatment of choice for large proximal ureteral stones [35].

Drake et al. reported the advantages and disadvantages of URSL compared with SWL in treating upper ureteral stones in a systematic review [33]. Of the total of 47 studies included, 22 studies compared URSL and SWL. The results showed that URSL is related to a significantly greater SFR up to 4 weeks, but not at 3 months. URSL was also associated with fewer retreatments but with greater complications and longer hospital stays. The authors concluded that both URSL and SWL are safe and effective and that treatment should be provided according to the patients’ environment and preferences [33]. However, this study had a limitation that of these twenty-two studies assessed, only four were RCTs, one was a quasi-RCT, and seventeen were nonrandomized studies.

Matlaga et al. reported that URSL is more efficacious than SWL for distal ureter stones [36]. In the 2012 Cochrane meta-analysis, URSL showed a higher SFR, but a greater complication rate, and longer hospital stays than SWL [37]. The authors suggest that these results are due to marked heterogeneity in the type of SWL device, size of the ureteroscope, type of intracorporeal lithotripter, and surgeons’ proficiency in the included studies. The evidence does not clearly suggest what treatment would be good [37].

In our current study, URSL showed a greater SFR than SWL regardless of the size of the ureteral stones. This change was significant in RCT studies (*p* < 0.01, OR = 0.40, 95% CI 0.30–0.55, I² = 29%) and in non-RCT studies (*p* < 0.01, OR = 0.33, 95% CI 0.21–0.52, I² = 83%) (Figure 3). Additionally, URSL showed in our study that the need for retreatment procedure was significantly lower, regardless of the size of the ureteral stones compared to SWL (*p* < 0.01, OR = 10.22, 95% CI 6.76–15.43, I² = 54%) (Figure 4).

A limitation of our meta-analysis is that only five randomized studies were included; the remainder of the studies were retrospective and had a high heterogeneity. Another limitation is that we did not analyze according to the location of the ureteral stone. Additionally, there were differences in each study; for example, the type of SWL devices, the type of ureteroscope and lithotripsy devices, and the experience of the surgeon. Additional high-quality RCTs are required to make more appropriate comparisons between URSL and SWL for ureteral stones.

## 5. Conclusions

Regardless of the size of the ureteral stones, URSL was related to a higher SFR than SWL. In comparison, SWL was related to a higher retreatment rate than URSL in our meta-analysis. However, more randomized trials are required to identify definitive conclusions.

## Figures and Tables

**Table 1:** Characteristics of included studies.

Author Year	Country	Design	Procedure	Stone Size (cm)	No. Patients	Mean Age	Quality Assessment (SIGN)
Dasgupta et al.	UK	RCT	ESWL	≤1	302	52.1	1+
2021 [23]			URSL		302	50	
Bahçeci et al.	Turkey	Retrospective	ESWL	<1	94	46	2+
2021 [22]				≥1	72		
			URSL	<1	213	46	
				≥1	403		
Kartal et al.	Turkey	Retrospective	ESWL	1.3 ± 0.3	162	43.6 ± 12.6	2+
2020 [25]			URSL	1.4 ± 0.3	119	43.9 ± 13.1	
			fURSL	1.4 ± 0.2	201	44.5 ± 13.1	
Rehman et al.	USA	RCT	ESWL	1.01 ± 0.2	75	41.2 ± 3.2	1-
2020 [26]			URSL	2.4 ± 0.2	75	40.9 ± 3.7	
Iqbal et al.	Pakistan	Retrospective	ESWL	1.0 ± 0.4	36	39.2 ± 13.4	2+
2018 [24]			URSL	1.4 ± 0.7	37	43.1 ± 13.7	
Cone et al.	USA	Retrospective	ESWL	<1.5	51	53 ± 14	2+
2017 [12]			URSL		62	54 ± 16	
Dell’Atti et al.	Italy	Retrospective	ESWL	≥1	313	46.2 ± 1.5	2+
2016 [13]			URSL		324	49.4 ± 2.1	
Khalil	Kuwait	Prospective	ESWL	<2	37	37.1 ± 8.8	2+
2013 [14]			URSL		45	35.2 ± 10.4	
Islam et al.	Pakistan	RCT	ESWL	<2.5	68	35.4 ± 9.2	2+
2012 [9]			URSL		68	35.3 ± 9.5	
Zhang et al.	China	RCT	ESWL	0.5–2.5	257	49	1+
2011 [10]			URSL		269	50	
Huang et al.	Taiwan	Prospective	ESWL	<1	201	52.5 ± 16.1	2+
2009 [15]				≥1	159		
			URSL	<1	40	49.5 ± 12.7	
				≥1	48		
Ziaee et al.	Iran	Prospective	ESWL	≥1	126	42.5	2+
2008 [16]			URSL		40	40.5	
Lee et al.	Taiwan	RCT	ESWL	≥1	22	54.2 ± 16.7	1-
2008 [11]			URSL		20	48.5 ± 13.3	
Wu et al.	Taiwan	Prospective	ESWL	<1	68	47.5 ± 1.5	2+
2005 [17]				≥1	51	51.5 ± 1.9	
			URSL	<1	45	51.0 ± 2.0	
				≥1	56	53.8 ± 1.5	
Wu et al.	Taiwan	Prospective	ESWL	≥1	41	51	2+
2004 [18]			URSL		39	51	
Zeng et al.	China	Prospective	ESWL	0.5–2.1	210	51	2-
2002 [19]			URSL		180	40	
Chang et al.	Taiwan	Retrospective	ESWL	<1	432	48.2	2-
2001 [20]				≥1	92		
			URSL	<1	381	48.3	
				≥1	49		
Yip et al.	Singapore	Retrospective	ESWL	<1	23	54	2-
1998 [21]				≥1	26		
			URSL	<1	34	47	
				≥1	27		

ESWL, extracorporeal shock wave lithotripsy; URSL, ureteroscopic lithotripsy; fURSL, flexible ureteroscopic lithotripsy; RCT, randomized controlled trials; SD, standard deviation. The quality assessment was indicated by Scottish Intercollegiate Guidelines Network (SIGN); 1+ means well-conducted RCT with a low risk of bias; 1- means RCT with a high risk of bias; 2+ means well-conducted cohort studies with a low risk of bias; 2- means cohort studies with a high risk of bias.

**Table 2:** MINORS score in nonrandomized studies included in the review.

	A Clearly Stated Aim	Inclusion of Consecutive Samples	Prospective Collection of Data	Endpoints Appropriate to the Aim of the Study	Unbiased Assessment of the Study Endpoint	Follow-up Period Appropriate to the Aim of the Study	Loss to Follow up Less than 5%	Prospective Calculation of the Study Size	An Adequate Control Group	Contemporary Groups	Baseline Equivalence of Groups	Adequate Statistical Analyses	Total
Bahçeci et al. 2021 [22]	2	2	2	2	0	2	2	0	2	2	1	2	19
Kartal et al. 2020 [25]	2	2	2	2	0	2	2	0	2	2	2	2	20
Iqbal et al. 2018 [24]	2	2	2	2	0	2	2	0	2	2	2	2	20
Cone et al. 2017 [12]	2	2	2	2	0	2	2	0	2	2	1	2	19
Dell’Atti et al. 2016 [13]	2	2	2	2	0	2	2	0	2	2	2	2	20
Khalil 2013 [14]	2	2	2	2	0	2	2	0	2	2	2	2	20
Huang et al. 2009 [15]	2	2	2	2	0	2	2	0	2	2	2	2	20
Ziaee et al. 2008 [16]	2	2	2	2	0	2	2	0	2	2	1	2	19
WU et al. 2005 [17]	2	2	2	2	0	2	2	0	2	2	1	2	19
Wu et al. 2004 [18]	2	2	2	2	0	2	2	0	2	2	1	2	19
Zeng et al. 2002 [19]	2	2	2	2	0	2	2	0	2	2	1	2	19
Chang et al. 2001 [20]	2	2	2	2	0	2	2	0	2	2	1	2	19
Yip et al. 1998 [21]	2	2	2	2	0	2	2	0	2	2	1	2	19

MINORS, methodological index for nonrandomized studies. The items are scored 0 (not reported), 1 (reported but inadequate), or 2 (reported and adequate). The global ideal score is 16 for non-comparative studies and 24 for comparative studies.

## Data Availability

The data presented in this study are available in the article.

## Supplementary Materials

The following are available online at https://www.mdpi.com/article/10.3390/medicina57121369/s1, Table S1: PRISMA Checklist.
